# Life satisfaction, psychological distress, compassion satisfaction and resilience: when the pleasure of helping others protects veterinary staff from emotional suffering

**DOI:** 10.1007/s11259-024-10510-0

**Published:** 2024-08-20

**Authors:** Maria Manuela Peixoto, Olga Cunha

**Affiliations:** 1 Center for Psychology, University of Porto, Porto, Portugal; 2 HEI-Lab: Human Environment Interaction Lab, Lusófona University, Porto, Portugal; 3 Faculdade de Psicologia e Ciências da Educação, Centro de Psicologia da Universidade do Porto, Universidade do Porto, Rua Alfredo Allen, s/n, Porto, 4200-135 Portugal

**Keywords:** Veterinary staff, Compassion satisfaction, Life satisfaction, Psychological distress, Resilience, Perceived social support

## Abstract

**Introduction:**

Individuals working in veterinary field suffer significantly from mental health problems, and research has extensively focused on psychological and work-related predictors of psychological distress. This study intended to approach psychological distress through a positive lens by investigating the predictive role of life satisfaction on psychological distress in veterinary staff, and the mediating effect of compassion satisfaction, resilience and perceived social support.

**Methodology:**

A total of 868 veterinary staff (i.e. veterinarians, veterinary nurses, veterinary assistants and veterinary administrative staff) completed a web-survey assessing life satisfaction, psychological distress, compassion satisfaction, resilience, and social support.

**Results:**

Life satisfaction negatively predicts psychological distress, and compassion satisfaction and resilience showed a mediation effect on the relationship between life satisfaction and psychological distress, with compassion satisfaction explaining 59% and resilience 6.4% of the effect of life satisfaction on psychological distress.

**Conclusion:**

Current data support the role of life satisfaction as a protective dimension on psychological distress within a broader sample population of veterinary staff in Portugal, highlighting the role of compassion satisfaction and resilience in contributing in minimising distress among veterinary staff.

## Introduction

Veterinary careers appear to attract individuals with elevated levels of compassion, i.e., the joy and sense of fulfilment derived from helping and caring for others (Brannick et al. 2015; Moore et al. 2014). Working in the veterinary field has been described as paradoxical labour, conceptualised as compassionate work that involves various compensations but also entails suffering (Polachek and Wallace 2018; Rohlf et al. 2022). Empirical literature extensively documented that working in the veterinary field is strongly and positively associated with mental health problems such as depression, anxiety, stress, burnout, secondary traumatic stress, compassion fatigue, suicidal ideation, and suicide risk, which have a detrimental impact on overall well-being and life satisfaction (e.g., Bartram and Baldwin 2010; Best et al. 2020; Dow et al., 2019; Gardner and Hini 2006; Kogan et al. 2020).

Life satisfaction has been negatively correlated to psychological distress (e.g., Busseri and Peck 2015). Within veterinary sample population, life satisfaction appears to be intertwined with various life dimensions, including job satisfaction (Kersebohm et al. 2017). Existing research on psychological distress in veterinary sample populations has primarily adopted a psychopathological perspective, focusing on risk factors and work-related predictors of depression, anxiety, stress and/or burnout, and particularly in the effective costs of burnout (Chapman et al. 2024; Neill et al. 2022; Steffey et al. 2023a; Steffey, Griffon, Risselada, Scharf, Steffey et al. 2023a, b). This study diverges from previous approaches by centering on protective factors – specifically, exploring how life satisfaction, compassion, resilience, and perceived social support can serve as buffers against psychological distress among veterinary staff.

### Life satisfaction

Life satisfaction, a component of subjective well-being, refers to the positive experience of one’s own life (Diener et al, 1985). Life satisfaction encompasses various dimensions, including satisfaction with interpersonal relationships. Research supports the connection between life satisfaction and perceived social support (Kasprzak 2010), indicating that individuals who perceive stronger social support from family, friends, and significant others tend to experience greater satisfaction with their own lives. Furthermore, life satisfaction has been negatively associated with psychological distress and psychopathological symptoms (e.g. Busseri and Peck 2015).

For veterinarians, life satisfaction is intertwined with job satisfaction, as well as contentment in areas such as family, leisure time, health, and standard of living (Kersebohm et al. 2017). In the context of job satisfaction, promoting life satisfaction in veterinary workplaces necessitates factors such as positive social interactions, the perception of valuable work, feelings of safety and security, and the belief in contributing to valuable and caring work (Bartram and Boniwell 2007; Stoewen 2016). Consequently, it is evident that the life satisfaction of veterinary staff is related to perceived social support, compassion satisfaction, and mental health.

### Psychological distress

Individuals working in the veterinary field are known to face significant challenges in terms of mental health, encompassing symptoms such as depression, anxiety, and stress (Clise et al. 2021). Additionally, they grapple with issues like compassion fatigue and burnout (Scotney et al. 2019; Smith 2016), and are at a higher risk of suicide (Nett et al. 2015). This global prevalence of mental health problems within the veterinary profession (Best et al. 2020; Deacon and Brough 2017; Nett et al. 2015; Perret et al. 2020) emphasizes the urgent need for a comprehensive understanding of these to facilitate mental health promotion and alleviate the psychological distress associated with the field.

Data from veterinary staff indicates a significant correlation between psychological distress, social support, and resilience. More resilient veterinary staff members tend to report higher satisfaction with their social support networks and experience lower levels of psychological distress (Perret et al. 2020). This connection underscores the importance of fostering resilience and enhancing social support systems to mitigate the challenges faced by individuals in the veterinary profession.

### Compassion satisfaction

It is hypothesised that individuals who demonstrate empathy and a profound commitment to assisting others, even in challenging circumstances, are likely to experience greater compassion satisfaction (Sacco and Copel 2018). Thus, compassion satisfaction refers to the enjoyment derived from one’s caring work, aligning with an empathic and nurturing way of doing it effectively (Stamm 2005), and is closely related to self-compassion (Rohlf et al. 2022).

Research on compassion satisfaction and its relationship with psychological distress has been extensively studied in the field of human care (e.g., Craig and Sprang 2010; Hunsaker et al. 2015; Kelly et al. 2015; Zhang et al. 2018) and more recently in the field of animal care (e.g., Ouedraogo et al., 2021; Scotney et al. 2019). Substantial evidence suggests that low levels of compassion satisfaction are significantly correlated with psychological distress in veterinary students (McArthur et al. 2017), as well as with compassion fatigue and burnout in veterinary nurses (Smith 2016). This implies that veterinary professionals experiencing psychological distress and mental health problems are more likely to exhibit lower levels of compassion satisfaction. Interestingly, a study involving veterinarians found a correlation between compassion satisfaction, psychological distress, and individual engagement, suggesting that individual engagement acts as a protective factor, buffering (Pizzolon et al. 2019).

### Resilience

Resilience refers to an individual’s capacity to adapt positively amidst challenging and adverse circumstances, finding meaning in the face of stressors in daily life (Resnick 2014). This attribute holds significant importance for the enhancement and development of veterinary staff and teams (Cake et al. 2017; Kerrigan 2018; Taylor et al. 2022) to achieve well-being, life satisfaction, and good mental health outcomes (Taylor et al. 2022). Resilience, as a psychological dimension, has consistently demonstrated a positive association with psychological well-being (Campion 2020; Resnick 2014; Taylor et al. 2022).

In the realm of veterinary work, resilience is recognized as a protective factor against mental health problems (Campion 2020) and occupational stress (Lloyd and Campion 2017). It is also considered a valuable coping strategy for integration into prevention and intervention programs addressing mental health problems within this professional community (Taylor et al. 2022). Given the inherent daily stressors and adversities associated with veterinary work (Clise et al. 2021), the promotion of resilience as a coping strategy becomes particularly beneficial in preventing compassion fatigue and other mental health problems (Kerrigan 2018; Taylor et al. 2022). However, it is important to recognize that resilience per se is not a guarantee of good mental health outcomes, as it is a skill that can be fostered along with others related to self-care strategies (Lloyd and Campion 2017) and personal and/or contextual resources (Cake et al. 2017).

### Social support

Social support can be defined as the willingness of others to offer support, care, and help during difficult times, crises, and stressful situations (Sarason et al. 1983; Zimet et al. 1988). This multidimensional construct involves emotional, instrumental, and knowledge-based help from various individuals, including family, friends, and significant others (Carvalho et al. 2011; Zimet et al. 1988). Social support is based on stable and gratifying interpersonal interactions and relationships, closely related to effective coping with difficult situations and problems (Kahn and Nutter 2005). Moreover, research has shown that the positive perception of social support holds greater significance than the social support itself (Carvalho et al. 2011; Khawaja and Dempsey 2007). Furthermore, social support appears to be negatively related to psychological distress among individuals who have experienced traumatic situations. While the specific mechanisms by which social support protects against psychopathology remain unclear, research points to the possibility that self-compassion may function as a protective factor (Maheux and Price 2016).

### Current study

The veterinary staff are known to grapple with significant psychological distress, with existing research primarily focusing on risk factors and negative predictors of psychopathology. The current study seeks to adopt a positive approach to understanding psychological distress by exploring the predictive role of life satisfaction among a broader sample population of Portuguese veterinary staff (i.e. veterinarians, veterinary nurses, veterinary assistants, and veterinary administrative staff). The research also aims to investigate the mediating effect of compassion satisfaction, resilience, and perceived social support on the relationship between life satisfaction and psychological distress. It is expected that veterinary staff who are more satisfied with their own lives will experience lower levels of psychological distress and that higher compassion satisfaction, greater resilience, and greater perceived social support will act as a buffer against psychological distress.

## Method

### Procedures

This study is part of a larger project on mental health in veterinary staff, which received approval from the Ethics Committee from Lusofona University of Porto. The Portuguese versions of self-report measures were authorized for use in alignment with the study’s objectives. A web-survey addressing mental health within the veterinary community was disseminated via social media platforms, including Meta and LinkedIn, veterinary platforms and associations, and mailing lists from veterinary institutions (veterinary medical board, veterinary nurses association, among others), spanning from December 2022 to March 2023. Participants received the link for the web-survey along with a description of the study purpose, including information on confidentiality and anonymity of their participation. No personal data allowing identification was requested, and no IP addresses were recorded. Considering the sensitive nature of the study, a contact from the national health service for crisis psychological intervention was made available, along with the email address of the principal researcher. Participants were invited to participate after reading all the information about the study and were required to provide informed consent to proceed. Participation was entirely voluntary, and no incentives, monetary rewards, or prizes were offered. Inclusion criteria for the current study was 18 years or more and currently working in the medical veterinary field (i.e., veterinarian, veterinary nurse, veterinary assistant, veterinary administrative staff).

### Measures

#### Socio-demographic information

A questionnaire was developed for this study. Participants were asked to provide sociodemographic information, including age, sex, nationality, relationship status, academic degree, main activity at work, current psychiatric history and psychiatric medication, and current mental health treatment.

#### Life satisfaction

For assessing life satisfaction, participants answered to the Satisfaction with Life Scale (Diener et al. 1985) that included five statements that are classified according to a 7-point Likert scale from 1 (*strongly disagree*) to 7 (*strongly agree*). Score was computed by summing all items. Both original (Diener et al. 1985) and Portuguese versions (Simões 1992) achieved one-factor structure and yielded good psychometric properties.

#### Psychological distress

The Depression Anxiety Stress Scale − 21 items (DASS-21; Lovibond and Lovibond 1995) was used for measuring psychological distress, and includes 21 items that assess symptoms of depression, anxiety and stress. Participants rate the items using a 4-point *Likert* scale, from 0 (“*not at all*”) to 3 (“*most of the time*”), score was computed by summing all items, and multiplying by two to provide scores compatible with the DASS − 42 items (Lovibond and Lovibond 1995), and higher scores indicate more psychological distress. Both original (Lovibond and Lovibond 1995) and Portuguese version (Pais-Ribeiro et al. 2004) of the DASS-21 achieved a three-factor structure and yielded good psychometric properties.

#### Compassion satisfaction

 For assessing satisfaction with compassion, a subscale from the Professional Quality of Life Scale - Version 5 (PROQOL-5; Stamm 2009) was used. The PROQOL-5 allows to measure Compassion Satisfaction, Burnout, and Secondary Traumatic Stress within the professional context and comprises 30 items rated on a 5-point *Likert* scale, from 1 (“*never*”) to 5 (“*very often*”). Compassion satisfaction scoring was computed by summing the items of the scale, with higher scores suggesting greater satisfaction with compassion. For this study purpose, participants were asked to report to their animal patients and their owners. Both original and Portuguese versions of the PROQOL-5 identified three factors and yielded good psychometric properties (Stamm 2009).

#### Resilience

 The Brief Resilience Scale (Smith et al. 2008) was used for assessing resilience, and included six items answered according to a 5-point Likert scale from 1 (*strongly disagree*) to 5 (*strongly agree*). The total score was computed by summing all items after recoded inverted items, and higher scores suggested greater levels of resilience. Both original (Smith et al. 2008) and Portuguese versions (Almeida et al. 2023) achieved one-factor structure and yielded good psychometric properties.

#### Social support

Perceived social support was measured with the Multidimensional Scale of Perceived Social Support (MSPSS; Zimet et al. 1988), that included 12 items for measuring perceived adequacy of social support from family, friends, and significant others, rated with a 7-point Likert scale from 1 (*strongly disagree*) to 7 (*strongly agree*). Total score was computed by summing all items, with higher scores indicating greater perception of social support. Both original (Zimet et al. 1988) and Portuguese versions (Carvalho et al. 2011) achieved a three-factor structure and yielded good psychometric properties.

### Statistical plan

Data was processed and analysed using IBM SPSS version 29.0 software. Descriptive statistics were conducted for sample population and variables characterization, and Pearson’s coefficients correlations were calculated to determine the association between variables. Mediation analysis was performed with PROCESS macro 4.2, model 4 (Hayes 2022), and the mediational model included a regression equation between life satisfaction on psychological distress, through the mediation of compassion satisfaction, resilience, and social support, controlling for professional category and current psychiatric history. Direct and indirect effects on how life satisfaction predicts psychological distress were tested (Hayes 2022), and 5000 bootstrap samples based on 95% Bias-Corrected Bootstrap Confidence Intervals (95% BCBCI) were computed for assessing the mediation effects (Preacher and Hayes 2008). Percentage of mediation effect was estimated (Shrout and Bolger 2002), and mediation effect size was interpreted based on Cohen’s work (1988): null − 0.00, small − 0.14; medium − 0.39; and large − 0.59 (Farichild et al., 2009).

## Results

### Participants

This study consisted of a sample of 868 participants working in the veterinary field, with 457 (52.7%) veterinarians, 298 (34.3%) veterinary nurses, 104 (12.0%) veterinary assistants, and 9 (1.0%) veterinary administrative staff. The average age was 31.8 (*SD* = 7.4), ranging between 19 and 66 years. Table 1 depicts sociodemographic characterization of the sample.

**Table 1:** Sociodemographic characterization of the sample (*N* = 868)

Variables	*n*	%
Sex		
Men	133	15.3
Women	735	84.7
Nationality		
Portuguese	858	98.8
Other	10	1.2
Relationship status		
Single	553	63.7
Married/Civil union	296	34.1
Divorced/Widowed	19	2.2
Academic degree		
High school	50	5.8
Technical (post high school)	36	4.1
Bachelor	370	42.6
Master	410	47.2
Doctorate	2	0.2
Main activity		
Small animal	766	88.2
Exotic animal	9	1.0
Farm animal	12	1.4
City council	46	5.3
Other	35	4.0
Current psychiatric history		
Yes	232	26.7
No	636	73.3
Current psychiatric medication		
Yes	146	16.8
No	722	83.2
Current mental health treatment		
Yes	230	26.5
No	638	73.5

### Internal consistency

Internal consistency, for the current study, for the Satisfaction with Life Scale was 0.89, for the Depression Anxiety Stress Scale − 21 was 0.95, for the Compassion Satisfaction subscale was 0.88, for the Brief Resilience Scale was 0.60, and for the Multidimensional Scale of Perceived Social Support was 0.94.

### Pearson correlation coefficients

Table 2 illustrates the means, standard deviations, and ranges of all variables in study, and presented Pearson correlation coefficients.

**Table 2:** Means, standard-deviations, and Pearson’s correlation coefficients between all variables in study (*N* = 868)

Variables	M(SD)	1.	2.	3.	4.	5.
1. Life satisfaction	14.29 (4.72)	1.00				
2. Psychological distress	49.12 (29.73)	− 0.52***	1.00			
3. Compassion satisfaction	34.55 (6.79)	0.56***	− 0.36***	1.00		
4. Resilience	17.57 (3.86)	0.41***	− 0.45***	0.41***	1.00	
5. Social support	66.68 (16.31)	0.45***	− 0.28***	0.33***	0.23***	1.00

*** *p* <.001

### Mediation model

**Fig. 1:**
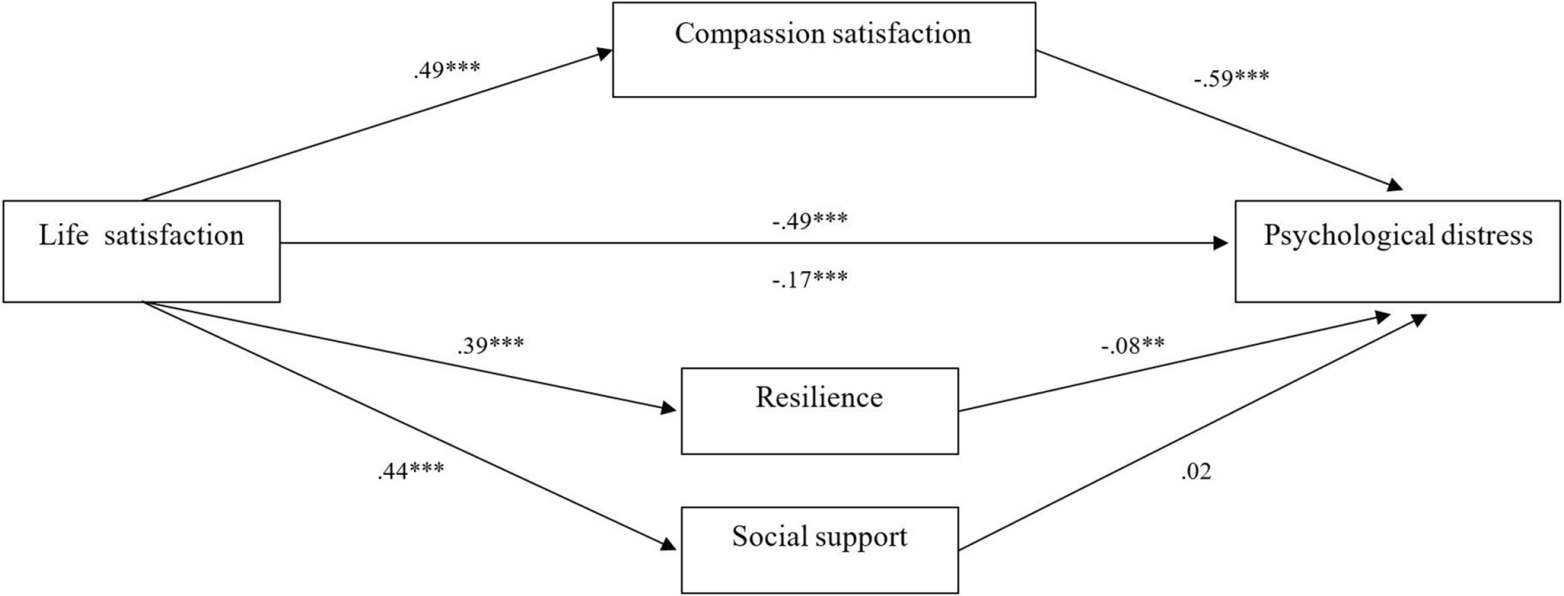
Mediation model of compassion satisfaction, resilience, and social support in the relationship between life satisfaction and psychological distress, controlling for professional category and current psychiatric history (*N* = 868).  Footnote: ** *p*  <.01; *** *p*  <.001; Compassion satisfaction mediated 59.0% of the total effect of life satisfaction on psychological distress, effect size = 0.29; Resilience mediated 6.4% of the total effect of life satisfaction on psychological distress, effect size = 0.03

The mediation model of life satisfaction predicting psychological distress, with compassion satisfaction, resilience, and social support as mediator variables, explained 58.0% of the variance in psychological distress, which was significant, *R*^*2*^ = 0.6, *F*(6,861) = 198.9, *p* <.001. The direct effect of life satisfaction on psychological distress was statistically significant, *β* = − 0.5, SE = 0.2, *t* = -16.9, *p* <.001; 95% BCBCI − 3.4– -2.7, controlling for professional category (*p* =.527) and for current psychiatric history (*p* <.001). The regression equation of life satisfaction on compassion satisfaction (mediator) was statistically significant, *β* = 0.5, SE = 0.1, *t* = 16.9, *p* <.001; 95% BCBCI 1.1–1.3, and the regression equation of compassion satisfaction on psychological distress was statistically significant, *β* = − 0.6, SE = 0.1, *t* = -21.3, *p* <.001; 95% BCBCI − 1.7– -1.4. Also, the regression equation of life satisfaction on resilience (mediator) was statistically significant, *β* = 0.4, SE = 0.0, *t* = 12.5, *p* <.001; 95% BCBCI 0.3–0.4, and the regression equation of resilience on psychological distress was statistically significant, *β* = − 0.1, SE = 0.2, *t* = -3.1, *p* =.002; 95% BCBCI − 1.0– -0.2. In addition, the regression equation of life satisfaction on social support (mediator) was statistically significant, *β*  =  0.4, SE = 0.1, *t* = 14.3, *p* <.001; 95% BCBCI 1.3–1.7, but the regression equation of social support on psychological distress was not statistically significant, *p* =.478. Finally, the regression equation of life satisfaction on psychological distress was statistically significant after controlling for mediators, *β* = − 0.2; SE = 0.2, *t* = -6.1, *p* <.001; 95% BCBCI − 1.4– -0.7 (Fig. 1).

## Discussion

The aim of the current study was to explore the predictive role of life satisfaction on psychological distress within a larger sample population of Portuguese veterinary staff, encompassing veterinarians, veterinary nurses, veterinary assistants, and veterinary administrative staff. Additionally, the study aimed to investigate the mediating effect of compassion satisfaction, resilience, and perceived social support in this association. As expected, the findings revealed that life satisfaction was a negative and statistically significant predictor of psychological distress. This suggests that veterinary staff experiencing higher levels of life satisfaction tend to have lower levels of psychological distress. Regarding mediating effects, only compassion satisfaction and resilience exhibited statistically significant effects on the relationship between life satisfaction and psychological distress, serving as buffers against psychological distress. Compassion satisfaction explained 59% of this relationship, while resilience explained 6.4%. Notably, no statistically significant mediating effect was observed for perceived social support.

Regarding the Pearson coefficient correlations among life satisfaction, psychological distress, compassion satisfaction, resilience, and perceived social support, all variables exhibited statistically significant correlations, aligning with expectations. Psychological distress displayed a negative correlation with life satisfaction, compassion satisfaction, resilience, and perceived social support, while all other variables demonstrated positive correlations with each other. These findings are consistent with previous research, which has consistently reported a negative relationship between psychological distress and life satisfaction (e.g. Busseri and Peck 2015), compassion satisfaction (e.g. McArthur et al. 2017), resilience and perceived social support (e.g. Perret et al. 2020). Moreover, a positive relationship was found between life satisfaction and compassion satisfaction (e.g., Bartram and Boniwell 2007; Stoewen 2016), resilience (e.g., Taylor et al. 2022) and perceived social support (e.g., Kasprzak 2010). These findings reinforce the notion that veterinary staff experiencing higher psychological distress tend to report lower life satisfaction, reduced resilience, a perceived lack of social support from their family, friends, and significant others, and diminished levels of compassion satisfaction. Notably, in the current study, the strongest Pearson coefficient correlations were observed between life satisfaction and compassion satisfaction (positive), and between life satisfaction and psychological distress (negative), followed by life satisfaction and social support, life satisfaction and resilience, resilience and psychological distress, and resilience and compassion satisfaction. Accordingly, compassion satisfaction and psychological distress emerged as particularly relevant factors influencing life satisfaction within the veterinary community. This suggests that efforts to enhance psychological well-being and increase compassion satisfaction may significantly contribute to the life satisfaction and overall well-being of individuals working in the veterinary field.

The primary finding of the current study underscores the mediating effect of compassion satisfaction and resilience in the relationship between life satisfaction and psychological distress within the veterinary community. As previously mentioned, heightened life satisfaction correlated with reduced levels of psychological distress among veterinary staff. This relationship was partially mediated by compassion satisfaction and resilience, which accounted for 65.4% of the effect of life satisfaction on psychological distress, with a medium mediating effect of compassion satisfaction and a small mediating effect of resilience. It appears that greater satisfaction, fulfilment, and contentment across various dimensions in life (Diener, 1985), contribute to increased well-being, subsequently associated with lower levels of psychological distress in general. While multiple mechanisms may operate in this pathway, the current study supports two distinct protective mechanisms. On one hand, veterinary staff capable of experiencing greater compassion satisfaction, characterized by heightened empathy and a profound sense of caring and assistance for animals and their owners in need, even in adverse situations (Sacco and Copel 2018); and on the other hand, individuals who exhibit greater resilience and possess the ability to adapt positively and find meaning in their work amidst challenging and adverse circumstances (Resnick 2014). Contrary to our expectations, the perception of greater support, care, and help from others during difficult times and stressful situations (Sarason et al. 1983; Zimet et al. 1988) did not emerge as a mechanism linking life satisfaction to psychological distress. It is possible that perceived social support acts as a buffer against psychological distress in other contexts or in certain situations, but in this particular scenario, when dimensions such as compassion satisfaction and resilience are taken into account, social support lacks power. Also, individuals that are satisfied with their lives, that are resilient and have compassion satisfaction may underperceived social support, which can impact the results. Overall, these results suggested that, in the veterinary community, the way life satisfaction protects against psychological distress was through interindividual mechanisms such as compassion satisfaction and resilience as opposed to interindividual dimensions such as perceived social support.

### Limitations

Despite the relevance of the current study, some limitations are acknowledged. This study relies on a convenience sample population that may not accurately represent the veterinary community. The sample population was predominantly female (which reflects the nature of the veterinary workforce; FVE, 2023), with a greater representation of veterinarians compared to other staff, mostly working with small animals, which may also be a reflection of the veterinary working environment in Portugal. Regarding the type of veterinary work, the majority of the sample population works with small animals. While the recruitment method utilized a web survey to access participants nationwide, it may limit inclusion to individuals comfortable with online platforms. The assessment of psychological distress in the sample population was solely based on self-reported measures, lacking clinical and medical diagnosis through semi-structured interviews. Nevertheless, only reliable and validated self-report measures were used, allowing us to measure the theoretical constructs/variables in the current study in a trustworthy way for the sample population. It also offers the opportunity to compare our data with data from other sample populations and/or countries. Although the study covers symptomatology related to depression, anxiety, and stress, it does not provide information on burnout, compassion fatigue, or suicidal ideation. Also, the study does not delve into the frequency and intensity of traumatic stressful events. The scale that measures resilience achieved a threshold internal consistency value, which could also interfere with reliability of the results. In what concerns to the scale for assessing social support, it only measures perceived social support that includes family, friends and significant others, and did not allow to assess community social support or other relevant social support. With regard to other variables serving as potential buffers against psychological distress, the study focuses on compassion satisfaction, resilience, and perceived social support. However, other dimensions such as adaptive coping strategies, job satisfaction, and work-life balance should be considered in future research. Finally, to enhance the robustness of the findings, further studies are needed to overcome the identified limitations and replicate the current findings.

### Implications and future directions

Findings from the current study support the role of life satisfaction as a protective influence on psychological distress within a broader sample population of veterinary staff in Portugal. While this finding may not be surprising, it highlights the urgent need to prioritize and promote quality of life and well-being in this specific community to mitigate the heightened prevalence of mental health problems. Furthermore, the study highlights the distinctive roles played by compassion satisfaction and resilience as mechanisms influencing both life satisfaction and psychological distress. This novel finding supports the proposal to incorporate self-compassion and compassion-focused strategies into clinical interventions and mental health programs tailored for the veterinary community. To achieve this goal, according to Irons and Heriot-Maitland (2021), through the Compassionate Mind Training (8-week program) it is possible to enhance (self)compassion, positive emotions, well-being, and decrease distress. This program has revealed good psychological and physiological effects (Matos et al. 2017), inclusively, has revealed to be a reliable intervention for teachers, by diminishing levels of depression, stress and burnout, and for promoting levels of (self)compassion, compassion to others, psychological well-being and satisfaction with professional life (Matos et al. 2022). Additionally, it supports the implementation of programs aimed at enhancing coping strategies to foster resilience. Although this study is preliminary, it adds to a growing body of literature emphasising positive and protective factors that improve mental well-being. It is recommended that future research investigates the roles of protective factors in mitigating psychological distress among veterinary staff, addressing the limitations previously identified. Furthermore, it is recommended to develop and implement prevention and intervention programs that specifically target compassion, resilience, and life satisfaction, with a focus on evaluating their effectiveness in supporting the mental well-being of veterinary staff.

## Data Availability

No datasets were generated or analysed during the current study.

## References

[CR1] Almeida T, Caridade S, Cunha O (2023) The Brief Resilience Scale: Translation and adaptation to Portuguese (work in progress)

[CR2] Bartram DJ, Baldwin DS (2010) Veterinary surgeons and suicide: a structured review of possible influences on increased risk. Vet Rec 166:388–397. 10.1136/vr.b4794 20348468 10.1136/vr.b4794

[CR3] Bartram D, Boniwell I (2007) The science of happiness: achieving sustained psychological wellbeing. Pract 29:478–482. 10.1136/inpract.29.8.478

[CR4] Best CO, Perret JL, Hewson J, Khosa DK, Conlon PD, Jones-Bitton A (2020) A survey of veterinarian mental health and resilience in Ontario, Canada. Can Vet J 61(2):166–172 32020936 PMC6973204

[CR5] Brannick EM, DeWilde CA, Frey E, Gluckman TL, Keen JL, Larsen MR, Mont SL, Rosenbaum MD, Stafford JR, Helke KL (2015) Taking stock and making strides toward wellness in the veterinary workplace. J Am Vet Med Assoc 1;247(7):739– 42. 10.2460/javma.247.7.739 10.2460/javma.247.7.739 26383746

[CR6] Busseri MA, Peck E (2015) Do (even) depressed individuals believe that life gets better and better? The link between depression and subjective trajectories for life satisfaction. Clin Psychol Sci 3:715–725. 10.1177/2167702614547265

[CR7] Cake MA, McArthur MM, Matthew SM, Mansfield CF (2017) Finding the balance: uncovering resilience in the veterinary literature. J Med Vet Educ 44(1):95–105. 10.3138/jvme.0116-025R 10.3138/jvme.0116-025R 28206842

[CR8] Campion D (2020) Measuring resilience in veterinary practice. Vet Rec 2;186(15):486–488. 10.1136/vr.m1698 10.1136/vr.m1698 32358116

[CR9] Carvalho S, Pinto-Gouveia J, Pimentel P, Maia D, Pereira JM (2011) Características psicométricas da versão portuguesa da Escala Multidimensional De Suporte Social Percebido (Multidimensional Scale of Perceived Social Support - MSPSS). Psychologica 54:309–358. 10.14195/1647-8606_54_13

[CR10] Chapman AJ, Rohlf VI, Moser AY, Bennett PC (2024) Organizational factors affecting burnout in veterinary nurses: a systematic review. Anthrozoös, pp 1–36. 10.1080/08927936.2024.2333161

[CR11] Clise MH, Kirby N, McArthur ML (2021) Is veterinary work more than satisfying? A critical review of the literature. Vet Rec 188(10):e77. 10.1002/vetr.77 34018567 10.1002/vetr.77

[CR12] Cohen J (1988) Statistical power analysis for the behavioral sciences, 2nd edn. Lawrence Erlbaum Associates

[CR13] Craig CD, Sprang G (2010) Compassion satisfaction, compassion fatigue, and burnout in a national sample of trauma treatment therapists. Anxiety Stress Copin 23(3):319–339. 10.1080/10615800903085818 10.1080/10615800903085818 19590994

[CR14] Deacon RE, Brough P (2017) Veterinary nurses’ psychological well-being: the impact of patient suffering and death. Aust J Psychol 69(2):77–85. 10.1111/ajpy.12119

[CR15] Diener E, Emmons RA, Larsen RJ, Griffin S (1985) The satisfaction with Life Scale. J Pers Assess 49(1):71–75. 10.1207/s15327752jpa4901_13 16367493 10.1207/s15327752jpa4901_13

[CR16] Dow MQ, Chur-Hansen A, Hamood W, Edwards S (2019) Impact of dealing with bereaved clients on the psychological wellbeing of veterinarians. Aust Vet J 97:382–389. 10.1111/avj.12842 31364771 10.1111/avj.12842

[CR17] Fairchild AJ, Mackinnon DP, Taborga MP, Taylor AB (2009) R^2^ effect-size measures for mediation analysis. Behav Res Meth 41:486–498. 10.3758/BRM.41.2.486 10.3758/BRM.41.2.486 PMC2930209 19363189

[CR18] Federation of Veterinarians of Europe (2023) Survey of the Veterinary Profession in Europe 2023. Retrieved from: https://fve.org/cms/wp-content/uploads/FVE-Survey-2023_updated-v3.pdf

[CR19] Gardner DH, Hini D (2006) Work-related stress in the veterinary profession in New Zealand. N Z Vet J 54:119–124. 10.1080/00480169.2006.36623 16751842 10.1080/00480169.2006.36623

[CR20] Hayes AF (2022) Introduction to mediation, moderation, and conditional process analysis: a regression-based approach, 3rd edn. New York: The Guilford Press

[CR21] Hunsaker S, Chen HC, Maughan D, Heaston S (2015) Factors that influence the development of compassion fatigue, burnout, and compassion satisfaction in emergency department nurses. J Nurs Shcolars 47(2):186–194. 10.1111/jnu.12122 10.1111/jnu.12122 25644276

[CR22] Irons C, Heriot-Maitland C (2021) Compassionate mind training: an 8-week group for the general public. Psychol Psychother 94(3):443–463. 10.1111/papt.12320 33222375 10.1111/papt.12320

[CR23] Kahn H, Nutter CVJ (2005) Stress in veterinary surgeons: a review and pilot study. In: Antoniou ASG, Cooper CL (eds) Research Companion to Organizational Health Psychology. Edward Elgar, pp 293–303

[CR24] Kasprzak E (2010) Perceived social support and life-satisfaction. Pol Psychol Bull 41(4):144–154. 10.2478/v10059-010-0019-x

[CR25] Kelly L, Runge J, Spencer C (2015) Predictors of compassion fatigue and compassion satisfaction in acute care nurses. J Nurs Scholars 47(6):522–528. 10.1111/jnu.12162 10.1111/jnu.12162 26287741

[CR26] Kerrigan L (2018) The case for resilience in veterinary nursing care. Vet Nurse 9(8):396–400. 10.12968/vetn.2018.9.8.396

[CR27] Kersebohm JC, Lorenz T, Becher A, Doherr MG (2017) Factors related to work and life satisfaction of veterinary practitioners in Germany. Vet Rec Open 4(1):e000229. 10.1136/vetreco-2017-000229 29018534 10.1136/vetreco-2017-000229 PMC5623335

[CR28] Khawaja NG, Dempsey J (2007) Psychological distress in international university students: an Australian study. Aust J Guid Couns 17(1):13–27. 10.1375/ajgc.17.1.13

[CR29] Kogan LR, Wallace JE, Schoenfeld-Tacher R, Hellyer PW, Richards M (2020) Veterinary technicians and Occupational Burnout. Front Vet Sci 7. 10.3389/fvets.2020.00328 10.3389/fvets.2020.00328 PMC7303959 32596271

[CR30] Lloyd C, Campion DP (2017) Occupational stress and the importance of self-care and resilience: Focus on veterinary nursing. Irish Vet J 70;30. 10.1186/s13620-017-0108-7 29021894 10.1186/s13620-017-0108-7 PMC5613522

[CR31] Lovibond PF, Lovibond SH (1995) The structure of negative emotional states: comparison of the Depression anxiety stress scales (DASS) with the Beck Depression and anxiety inventories. Behav Res Ther 33(3):335–343. 10.1016/0005-7967(94)00075-U 7726811 10.1016/0005-7967(94)00075-u

[CR32] Maheux A, Price M (2016) The indirect effect of social support on post-trauma psychopathology via self-compassion. Pers Individ Dif 88:102–107. 10.1016/j.paid.2015.08.051

[CR33] Matos M, Duarte C, Duarte J, Pinto-Gouveia J, Petrocchi N, Basran J, Gilbert P (2017) Psychological and physiological effects of compassionate mind training: a pilot randomised controlled study. Mindfulness 8(6):1699–1712. 10.1007/s12671-017-0745-7

[CR34] Matos M, Palmeira L, Albuquerque I, Cunha M, Pedroso-Lima M, Galhardo A, Maratos FA, Gilbert P (2022) Building compassionate schools: pilot study of a compassionate mind training intervention to promote teachers’ well-being correction. Mindfulness 13(3):–799. 10.1007/s12671-022-01833-7

[CR35] McArthur ML, Andrews JR, Brand C, Hazel SJ (2017) The prevalence of compassion fatigue among veterinary students in Australia and the associated psychological factors. J Vet Med Educ 44(1):9–21. 10.3138/jvme.0116-016R3 28206848 10.3138/jvme.0116-016R3

[CR36] Moore IC, Coe JB, Adams CL, Conlon PD, Sargeant JM (2014) The role of veterinary team effectiveness in job satisfaction and burnout in companion animal veterinary clinics. J Am Vet Med Assoc 245(5):513–524. 10.2460/javma.245.5.513 25148093 10.2460/javma.245.5.513

[CR37] Neill CL, Hansen CR, Salois M (2022) The economic cost of burnout in veterinary medicine. Front Vet Sci 25(9):814104. 10.3389/fvets.2022.814104 10.3389/fvets.2022.814104 PMC8913590 35280150

[CR38] Nett RJ, Witte TK, Holzbauer SM, Elchos BL, Campagnolo ER, Musgrave KJ et al (2015) Risk factors for suicide, attitudes toward mental illness, and practice-related stressors among US veterinarians. J Am Vet Med Assoc 247(8):945–955. 10.2460/javma.247.8.945 26421408 10.2460/javma.247.8.945

[CR39] Ouedraogo FB, Lefebvre SL, Hansen CR, Brorsen BW (2021) Compassion satisfaction, burnout, and secondary traumatic stress among full-time veterinarians in the United States (2016–2018). J Am Vet Med Assoc 1;258(11):1259–1270. 10.2460/javma.258.11.1259 10.2460/javma.258.11.1259 33978434

[CR40] Pais-Ribeiro J, Honrado A, Leal I (2004) Contribuição para o estudo da adaptação portuguesa das escalas de ansiedade, depressão e stress (EADS) de 21 itens de Lovibond E Lovibond. Psicologia Saúde Doenças 5:229–239

[CR41] Perret JL, Best CO, Coe JB, Greer AL, Khosa DK, Jones-Bitton A (2020) Association of demographic, career, and lifestyle factors with resilience and association of resilience with mental health outcomes in veterinarians in Canada. J Am Vet Med Assoc 257(10):1057–1068. 10.2460/javma.2020.257.10.1057 33135980 10.2460/javma.2020.257.10.1057

[CR42] Pizzolon CN, Coe JB, Shaw JR (2019) Evaluation of team effectiveness and personal empathy for associations with professional quality of life and job satisfaction in companion animal practice personnel. J Am Vet Med Assoc 15(10):1204–1217. 10.2460/javma.254.10.1204 10.2460/javma.254.10.1204 31039097

[CR43] Polachek AJ, Wallace JE (2018) The paradox of compassionate work: a mixed-methods study of satisfying and fatiguing experiences of animal health care providers. Anxiety Stress Copin 31(2):228–243. 10.1080/10615806.2017.1392224 10.1080/10615806.2017.1392224 29064289

[CR44] Preacher KJ, Hayes AF (2008) Asymptotic and resampling strategies for assessing and comparing indirect effects in multiple mediator models. Behav Res Meth 40(3):879–891. 10.3758/BRM.40.3.879 10.3758/brm.40.3.879 18697684

[CR45] Resnick B (2014) Resilience in older adults. Top Geriatr Rehabil 30(3):155–163. 10.1097/TGR.0000000000000024

[CR46] Rohlf VI, Scotney R, Monaghan H, Bennett P (2022) Predictors of Professional Quality of Life in Veterinary professionals. J Vet Med Educ 49(3):372–381. 10.3138/jvme-2020-0144 34102096 10.3138/jvme-2020-0144

[CR47] Sacco TL, Copel LC (2018) Compassion satisfaction: a concept analysis in nursing. Nurs Forum 53(1):76–83. 10.1111/nuf.12213 28662300 10.1111/nuf.12213

[CR48] Sarason IG, Levine HM, Basham RB, Sarason BR (1983) Assessing social support: the Social Support Questionnaire. J Person Social Supp 44(1):127–139. 10.1037/0022-3514.44.1.127

[CR49] Scotney RL, McLaughlin D, Keates HL (2019) An investigation of the prevalence of compassion fatigue, compassion satisfaction and burnout in those working in animal-related occupations using the Professional Quality of Life (ProQoL) Scale. Vet Nurse 10(5):276–284

[CR50] Shrout PE, Bolger N (2002) Mediation in experimental and nonexperimental studies: new procedures and recommendations. Psychol Meth 7(4):422–445 12530702

[CR51] Simões A (1992) Ulterior validação de uma escala de satisfação com a vida (SWLS). Revista Portuguesa De Pedagogia 3:503–515

[CR52] Smith N (2016) A questionnaire based study to assess compassion fatigue in UK practising veterinary nurses. Vet Nurs 7(7):418–425

[CR53] Smith BW, Dalen J, Wiggins K, Tooley E, Christopher P, Bernard J (2008) The brief resilience scale: assessing the ability to bounce back. Int J Behav Med 15(3):194–200. 10.1080/10705500802222972 18696313 10.1080/10705500802222972

[CR54] Stamm B (2005) The professional quality of life scale: Compassion satisfaction, burnout & compassion fatigue/secondary trauma scales. Retrieved from: https://compassionfatigue.org/pages/ProQOLManualOct05.pdf

[CR55] Stamm BH (2009) Professional Quality of Life: Compassion Satisfaction and Fatigue– Version 5 (ProQOL). Retrieved from http://www.proqol.org/uploads/ProQOL_5_English.pdf

[CR56] Steffey MA, Griffon DJ, Risselada M, Buote NJ, Scharf VF, Zamprogno H, Winter AL (2023a) A narrative review of the physiology and health effects of burnout associated with veterinarian-pertinent occupational stressors. Front Vet Sci 3(10):1184525. 10.3389/fvets.2023.1184525 10.3389/fvets.2023.1184525 PMC10351608 37465277

[CR57] Steffey MA, Griffon DJ, Risselada M, Scharf VF, Buote NJ, Zamprogno H, Winter AL (2023b) Veterinarian burnout demographics and organizational impacts: a narrative review. Front Vet Sci 10:1184526. 10.3389/fvets.2023.1184526 37470072 10.3389/fvets.2023.1184526 PMC10352684

[CR58] Stoewen DL (2016) Veterinary happiness. Canad Vet J 57(5):539–541 27152045 PMC4827750

[CR59] Taylor DB, Johns KM, Reilly ML, Hedlefs RM (2022) A career development program: building resilience in veterinary undergraduates. Aust J Career Develop 31(1):26–41. 10.1177/10384162211066372

[CR60] Zhang YY, Han WL, Qin W, Yin HX, Zhang CF, Kong C, Wang YL (2018) Extent of compassion satisfaction, compassion fatigue and burnout in nursing: a meta-analysis. J Nurs Manag 26(7):810–819. 10.1111/jonm.12589 30129106 10.1111/jonm.12589

[CR61] Zimet GD, Dahlem N, Zimet S, Farley G (1988) The multidimensional scale of perceived social support. J Pers Assess 52:30–41. 10.1207/s15327752jpa5201_2

